# Complete Genome Sequence of *Mycobacterium chimaera* SJ42, a Nonoutbreak Strain from an Immunocompromised Patient with Pulmonary Disease

**DOI:** 10.1128/genomeA.00963-17

**Published:** 2017-09-14

**Authors:** Nabeeh A. Hasan, René L. Warren, L. Elaine Epperson, Allyson Malecha, David C. Alexander, Christine Y. Turenne, Daniel MacMillan, Inanc Birol, Stephen Pleasance, Robin Coope, Steven J. M. Jones, Marc G. Romney, Monica Ng, Tracy Chan, Mabel Rodrigues, Patrick Tang, Jennifer L. Gardy, Michael Strong

**Affiliations:** a Center for Genes, Environment and Health, National Jewish Health, Denver, Colorado, USA; b Genome Sciences Centre, BC Cancer Agency, Vancouver, British Columbia, Canada; c Cadham Provincial Laboratory, Winnipeg, Manitoba, Canada; d Diagnostic Services Manitoba, Winnipeg, Manitoba, Canada; e Providence Health Care, Vancouver, British Columbia, Canada; f St. Paul’s Hospital, Vancouver, British Columbia, Canada; g BC Centre for Disease Control, Vancouver, British Columbia, Canada; h School of Population and Public Health, UBC, Vancouver, British Columbia, Canada

## Abstract

*Mycobacterium chimaera*, a nontuberculous mycobacterium (NTM) belonging to the *Mycobacterium avium* complex (MAC), is an opportunistic pathogen that can cause respiratory and disseminated disease. We report the complete genome sequence of a strain, SJ42, isolated from an immunocompromised male presenting with MAC pneumonia, assembled from Illumina and Oxford Nanopore data.

## GENOME ANNOUNCEMENT

*Mycobacterium chimaera* is an emerging pathogen causing pulmonary and disseminated infections, especially in patients with underlying respiratory conditions or those who are immunocompromised. With recent *M. chimaera* infections linked to exposure to contaminated heater-cooler unit (HCU) devices during open-chest surgery (1), an increasing number of outbreak-associated genomes are available (2–8). However, few nonoutbreak genomes representing *M. chimaera* clinical isolates exist (9, 10). We report the genome sequence of SJ42, a clinical isolate from an HIV-positive patient presenting with *Mycobacterium avium* complex (MAC) pneumonia and no history of HCU exposure.

We grew SJ42 from a sputum sample on Lowenstein-Jensen slants (Bio-Media, Woodbridge, Ontario, Canada) at 37°C for 4 weeks before extracting genomic DNA using Qiagen’s MagAttract HMW DNA kit. For nanopore sequencing, DNA was sheared to ∼8 kb in a Covaris g-TUBE and then subjected to PreCR formalin-fixed paraffin-embedded (FFPE) repair, end repair, and A-tailing with New England Biolab’s NEBNext reagents. Two-dimensional sequencing adapters were added, and the library was purified using Oxford Nanopore’s 2D Genomic DNA kit (catalog number SQK-LSK208). The library was sequenced on a MinION Mk1B with SpotON Flow Cell R9.4 using a standard 48-hour 2D sequencing run with cloud basecalling. DNA was also sequenced on an Illumina MiSeq instrument using the Nextera library preparation protocol and MiSeq reagent kit v3 (Illumina, San Diego, CA, USA).

Nanopore sequence reads were filtered using Nanopolish v0.5.0 and assembled *de novo* concurrently with MiSeq reads using Unicycler (11). The assembly was polished with unicycler_polish using all sequence reads and scaffolded with LINKS v1.8.5 (12) using nanopore reads. Assembly gaps were closed with Sealer v1.5.2 using MiSeq reads exclusively (13). The resulting assembly was aligned against other *M. chimaera* genomes using MAUVE 2.3.1 (14). Genomic features were identified and annotated using the NCBI Prokaryotic Genome Automatic Annotation Pipeline. We conducted core genome comparisons with Roary (15).

The *M. chimaera* SJ42 genome consists of three scaffolds, equaling 5,937,236 bp (a 5,891,694-bp chromosome, 33,560-bp plasmid, and 13,458-bp plasmid) and a G+C content of 67.52%. We predicted 5,696 coding sequences, including 5,517 protein-coding genes and 242 pseudogenes. Our assembly contains 52 tRNAs, 3 noncoding RNAs (ncRNAs), and 1 rRNA cistron consisting of the 16S, 23S, and 5S rRNA genes.

Whole-genome comparisons of *M. chimaera* SJ42 to AH16 (GenBank accession number CP012885) (9); MCIMRL6, MCIMRL4, and MCIMRL2 (LJHN00000000, LJHM00000000, and LJHL00000000) (10); MC ANZ045 (NZ_LT703505) (16); FI-0169 (NZ_MRBR00000000) (2); JCM 14737 (NZ_MNAM00000000) (5); and CDC2015-22-71 revealed phylogenetic distances between 34,471 single nucleotide polymorphisms (SNPs) (MC ANZ045) and 37,539 SNPs (AH16). The nine genomes shared a core gene set of 4,201 genes and had an average nucleotide identity (ANI) of ≥97.68% (17).

SJ42 is the first *M. chimaera* genome from an immunocompromised patient presenting with MAC pneumonia and is phylogenetically distinct from previously sequenced strains. The SJ42 genome will serve as a resource for epidemiological investigations of *M. chimaera* infections.

### Accession number(s).

The SJ42 assembly was deposited in DDBJ/EMBL/GenBank under the accession numbers CP022223 to CP022225, with MinION and Illumina raw data under BioProject number PRJNA391747 and BioSample number SAMN07274465.
